# Mastering organismal aging through the endoplasmic reticulum proteostasis network

**DOI:** 10.1111/acel.13265

**Published:** 2020-10-31

**Authors:** Rebecca C. Taylor, Claudio Hetz

**Affiliations:** ^1^ MRC Laboratory of Molecular Biology Cambridge UK; ^2^ Center for Geroscience Brain Health and Metabolism Santiago Chile; ^3^ Biomedical Neuroscience Institute Faculty of Medicine University of Chile Santiago Chile; ^4^ Program of Cellular and Molecular Biology Institute of Biomedical Sciences University of Chile Santiago Chile; ^5^ Buck Institute for Research on Aging Novato CA USA

**Keywords:** aging, autophagy, cell‐nonautonomous, ER stress, protein misfolding, proteostasis

## Abstract

The aging process is characterized by a progressive decline in the function of most tissues, representing the main risk factor in the development of a variety of human diseases. Studies in multiple animal models have demonstrated that interventions that improve the capacity to maintain endoplasmic reticulum (ER) proteostasis prolong life and healthspan. ER stress is monitored by the unfolded protein response (UPR), a signaling pathway that mediates adaptive processes to restore proteostasis or the elimination of damaged cells by apoptosis. Here, we discuss recent advances in understanding the significance of the UPR to aging and its implications for the maintenance of cell physiology of various cell types and organs. The possible benefits of targeting the UPR to extend healthspan and reduce the risk of developing age‐related diseases are also discussed.

## Graphical abstract

Crosstalk between aging pathways and the UPR. The interrelation between signaling pathways that regulate aging and the UPR is indicated, with key components and intervention strategies that modify the aging process.

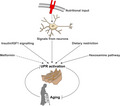

## 1 INTRODUCTION

“Every man desires to live long, but no man wishes to be old.” So said Jonathan Swift, and with good reason. Aging is not only a chronological process of accumulating years, but is also “a persistent decline in the age‐specific fitness components of an organism due to internal physiological deterioration” (Rose, 1991). Increased mortality with age reflects both increased frailty and a greater susceptibility to a wide range of diseases. These include neurodegenerative conditions such as Alzheimer's, Parkinson's, and Huntington's diseases, as well as cancer, cardiovascular disease, and metabolic illnesses such as type 2 diabetes. Much research has been devoted to the underlying causes of this age‐associated disease susceptibility and to the possibility that aging itself could be delayed or even reversed, reducing vulnerability to a range of serious conditions simultaneously. While aging has in the past frequently been thought of as an inevitable process of gradual breakdown, akin to the mechanical failure of a car or other machine, studies of the evolutionary origins of aging have suggested that the true nature of the process is more complex, involving tradeoffs between somatic maintenance and reproduction, accumulation of deleterious late‐acting mutations, and the negative consequences of genes and molecular pathways that act beneficially in early life (Kirkwood & Austad, 2000). Furthermore, investigations in model organisms have revealed that the lifespan of a species is not set in stone but can in fact be modulated, through both environmental changes and genetic alterations of specific signaling pathways. Dietary restriction, reduced insulin signaling levels, and changes to respiratory rates are among the modulations that can extend longevity in multiple species (Fontana et al., 2010).

Exploring the means by which these interventions prolong longevity has revealed universal features of the aging process, as well as common mechanisms upon which different treatments converge to promote improved aging phenotypes. Older cells suffer from a decline in the maintenance of organellar, metabolic, and protein homeostasis, for example, leading to accumulations of toxic or damaged organelles and macromolecules that likely contribute to reduced cellular fitness (Pomatto & Davies, 2017). Altered activity of the pathways that influence the aging process frequently delays the appearance of these hallmarks of aging, postponing the age‐associated loss of cellular homeostasis. This suggests the exciting possibility that intervention in the activity of these pathways, or direct manipulation of the mechanisms that preserve cellular homeostasis, may represent a means to delay or treat human conditions of aging.

### 1.1 Proteostasis impairment in aging

The idea that aging is the main risk factor to develop a variety of human diseases suggests that the mechanisms that govern the normal aging process may contribute to disease etiology or determine the threshold of cellular disturbance to trigger a pathological condition (Campisi et al., 2019). The accumulation of abnormal protein aggregates is a salient feature of a variety of age‐related diseases, in particular neurodegenerative conditions such as Parkinson's, Alzheimer's, ALS, and Prion‐related disorders (Soto & Pritzkow, 2018). In most of these conditions, the protein misfolding process occurs in the absence of genetic mutations to the proteins accumulated, suggesting that suboptimal function of the pathways that sustain the production and quality control of proteins are altered (Kaushik & Cuervo, 2015). In fact, studies in multiple model organisms suggest that a reduction in the buffering capacity of the proteostasis network is one of the fundamental pillars of aging and may explain in part the accumulation of abnormal protein aggregates during aging or the manifestation of genetic diseases where the mutant protein is expressed from development but the disease manifests only in the elderly.

Proteostasis is maintained through the dynamic integration of all the processes that control the production of proteins (Balch et al., 2008). These include highly complex pathways mediating protein translation, folding, protein maturation, trafficking, degradation, and targeting to the final destination. Each of these processes involves specialized components that are regulated at different levels and can be compartmentalized to specific organelles or membrane subdomains. Protein quality control mechanisms ensure the proper folding of proteins or their degradation through the proteasome and the lysosomes. Several routes deliver substrates to the lysosome including the macroautophagy pathway, microautophagy, the endosomes, and chaperone‐mediated autophagy (Scrivo et al., 2018). For proteasome‐mediated degradation, cytosolic proteins are directly targeted through ubiquitination or are delivered from intracellular compartments such as the ER by the ER‐associated degradation (ERAD) pathway (Sun & Brodsky, 2019).

A variety of perturbations can alter the production of proteins inside the cell, and thus, specialized adaptive responses have evolved to cope with protein folding stress, regulating the expression of multiples components of the proteostasis network. Among them, the heat shock response (HSR), the integrated stress response (IRS), and the unfolded protein response (UPR) at the ER and mitochondria are the main feedback mechanisms to reduce the load of misfolded proteins inside the cell to recover proteostasis (Galluzzi et al., 2014; Powers & Balch, 2013). These stress reactions are governed by specialized sensors that signal to the cytosol and nucleus to reinforce existing mechanism to produce correctly folded proteins. Studies using simple model organisms suggest that the buffering capacity of the proteostasis network declines with aging. For example, an impaired induction of adaptive responses has been reported during aging in *Caenorhabditis* *elegans* when animals are exposed to exogenous agents that perturb the proteostasis network (i.e., ER stress, mitochondrial toxins, and heat shock) (reviewed in (Mardones et al., 2015)). Interestingly, functional studies using genetic manipulation of stress responses have demonstrated that the adaptive components of the ER‐UPR are fundamental to determine lifespan and healthspan in invertebrate models including yeast, flies, and worms (reviewed in (Martinez et al., 2017)) (see next sections). Furthermore, the activity of the UPR in neurons and the intestine has been shown to regulate whole organismal aging in worms. In this article, we review recent findings suggesting a central role of the UPR in controlling the aging process, in addition to the integration of whole animal proteostasis and metabolism through cell‐nonautonomous mechanisms. In the next section, we summarize most recent findings addressing the significance of ER proteostasis to aging and discuss possible links to various homeostatic pathways beyond protein folding stress that determine “when and how” an organism ages.

### 1.2 ER stress signaling and the UPR

ER homeostasis is constantly challenged by physiological demands that increase the secretory capacity of the cell. In fact, specialized secretory organs and cells (i.e., secretory glands, plasmatic B cells, exocrine, and endocrine pancreas) require an active UPR to sustain efficient protein production and avoid proteotoxicity (Hetz et al., 2020). Chronic ER stress is also observed in a variety of diseases including neurodegenerative conditions, metabolic syndromes, and cancer (Wang & Kaufman, 2016). In this context, different cellular perturbations are responsible for the induction of ER stress including the expression of mutant proteins that are cargo of the secretory pathway, inhibition of ERAD, or the activity of the proteasome, altered calcium homeostasis, and exacerbated levels of oxidative stress, among other cell injuries (Walter & Ron, 2011). To ensure protein folding fidelity and to maintain the secretory capacity of the cell, the UPR is engaged, constituted by parallel signaling networks that regulate mRNA translation and gene expression to reduce the load of misfolded proteins and restore cell function.

The main UPR pathways in mammals are mediated by three signaling cascades initiated by the ER‐located sensors IRE1α and β, PERK, and ATF6α and β. IRE1α is an ER localized kinase and endoribonuclease that under ER stress oligomerizes and autophosphorylates to engage its RNase domain (Karagoz et al., 2019). The activity of IRE1α catalyzes the excision of a small 26‐nucleotide intron from the mRNA encoding the transcription factor X‐box binding protein 1 (XBP1) in mammals and thereby shifts the translational open reading frame (Walter & Ron, 2011). Through this mechanism, an active transcription factor is expressed known as XBP1s (for the spliced form) that upregulates genes involved in protein folding, general secretion, and ERAD (Hetz et al., 2011). IRE1α can also cleave a restricted set of mRNAs and microRNAs, leading to their degradation, a process known as regulated IRE1‐dependent decay (RIDD) (Maurel et al., 2014). RIDD may contribute by reducing the levels of certain mRNAs to attenuate protein misfolding load in the ER, but also operates as a signaling event impacting multiple cellular process including sterile inflammation and apoptosis by regulating the expression of TXNIP, the inflammasome, caspase‐2, death receptors, and among others (Hetz & Papa, 2018). Additionally, IRE1α signals as a scaffold by associating with different adapter proteins and signaling molecules to crosstalk with other stress pathways through the formation a signaling platform referred to as the UPRosome (Hetz & Glimcher, 2009). This scaffold function of IRE1α has been linked to the regulation of various cellular processes including macroautophagy, mitochondrial metabolism, cytoskeleton dynamics, and MAP kinase signaling (see examples in (Carreras‐Sureda et al., 2019; Hetz, et al., 2020; Urra et al., 2018)).

**FIGURE 1:**
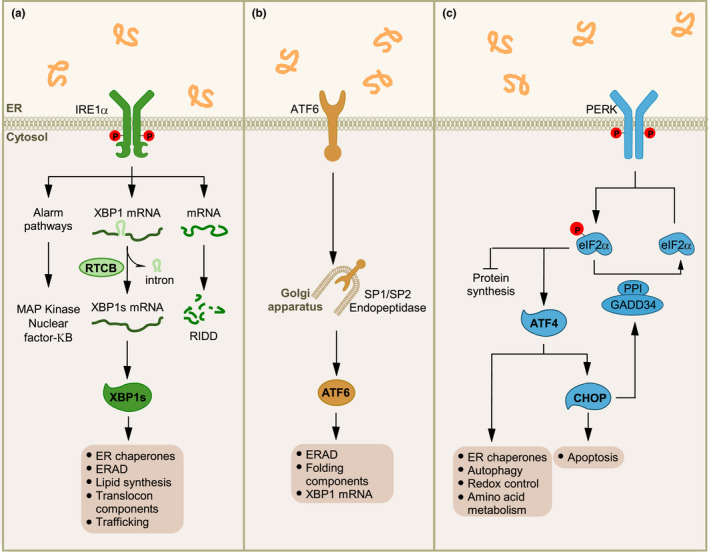
UPR signaling and cell death control. Under ER stress, three UPR signaling branches are activated. (a) The ER stress sensor IRE1α contains an RNase domain in the cytosol that splices *XBP1* mRNA, which encodes a potent transcription factor that activates expression of UPR target genes involved in ER proteostasis and cell pathophysiology. IRE1α RNase can also cleave ER‐associated mRNAs or noncoding functional RNAs, leading to their degradation through RIDD which modulates the protein folding load, cell metabolism, inflammation, and inflammasome signaling pathways. The IRE1α cytosolic domain may also serve as a scaffold to recruit adaptor proteins and signaling molecules. Further, the IRE1αUPR branch is involved in mitochondria‐dependent apoptosis through RIDD or activation of TRAF2‐JNK signaling. (b) ATF6 transits to the Golgi apparatus where it is cleaved by the proteases S1P and S2P, releasing an active cytosolic ATF6 fragment (ATF6p50). This fragment translocates to the nucleus, activating transcription of the UPR target genes involved in ERAD and folding. (c) Upon activation, the ER stress sensor PERK phosphorylates eukaryotic translation initiation factor eIF2α, reducing the overall frequency of mRNA translation initiation. However, the *ATF4* mRNA is preferentially translated in the presence of phosphorylated eIF2α. ATF4 activates the transcription of UPR target genes encoding factors involved in the antioxidant response, protein folding, amino acid biosynthesis, autophagy, and apoptosis. Irreversible ER stress triggers the expression of the pro‐apoptotic factor CHOP (transcription factor C/EBP homologous protein) and GADD34 via ATF4. GADD34 targets protein phosphatase 1 (PP1) to dephosphorylate eIF2α and thereby restore mRNA translation. CHOP promotes ER stress‐induced apoptosis by modulating members of the BCL‐2 or BH3‐only family, stimulating protein synthesis, and exacerbating protein folding defect

A rapid reaction under ER stress is also initiated by PERK, a kinase that attenuates the rate of protein synthesis by phosphorylating the α subunit of eukaryotic translation initiation factor 2 (eIF2α) (Costa‐Mattioli & Walter, 2020). This signaling event contributes to relieve ER stress by preventing the influx of newly synthesized proteins into the ER lumen. In addition, phosphorylation of eIF2α allows the translation of specific mRNAs that contain upstream open reading frame (uORF) sequences in their 5’ untranslated regions (Lu et al., 2004; Yaman et al., 2003), including the mRNA encoding the transcription factor ATF4. ATF4 has a dual role in the ER stress response because it regulates adaptive reactions by upregulating genes involved in redox homeostasis, amino acid metabolism, apoptosis, and autophagy, but also key apoptosis mediators (Pakos‐Zebrucka et al., 2016) (Figure 1). ATF4 also controls a feedback loop to dephosphorylate eIF2α and restore protein synthesis through upregulation of the phosphatase regulatory subunit GADD34 (growth arrest and DNA damage‐inducible 34) (Costa‐Mattioli & Walter, 2020).

Under ER stress, ATF6 transits from the ER to the Golgi apparatus, where it is cleaved by site‐1 (S1P) and site‐2 (S2P) proteases to release the cytosolic domain containing an active basic leucine zipper (bZIP) transcription factor. This fragment termed ATF6f or ATF6p50 translocates to the nucleus to upregulate the expression of genes gene expression (Glembotski et al., 2019). XBP1s and ATF6f also promote ER and Golgi biogenesis to augment the secretory capacity of the cell suffering from ER stress.

Several mechanisms are proposed to engage a terminal UPR response to eliminate cells suffering from chronic ER stress. Overall, a network of signaling events rather than a single pathway control cell demise upon irreversible damage (Figure 1). ER stress triggers the activation of the canonical mitochondrial apoptosis pathway, involving the conformational activation of the pro‐apoptotic members of the BCL‐2 family BAX and BAK at the mitochondria, resulting in the release of cytochrome c and the activation of the caspase cascade through the apoptosome (Urra et al., 2013). Upstream BH3‐only proteins of the BCL‐2 family are central factors that mediate ER stress‐induced apoptosis by engaging BAX and BAK at the mitochondrial membrane. Under ER stress, BH3‐only proteins are transcriptionally upregulated through ATF4/CHOP (i.e., PUMA, NOXA, and BIM) or are activated by post‐translational modifications (i.e., BID and BIM) (Pihan et al., 2017). The expression of ATF4 and CHOP increases oxidative stress and proteotoxicity by boosting protein synthesis in the stressed cell (Han et al., 2013; Marciniak et al., 2004). PERK signaling also upregulates the expression of death receptor 5 (DR5), which might directly bind misfolded proteins to induce its activation (Lam et al., 2020; Lu et al., 2014). Regulation of miRNAs, inflammatory signals, and sustained intracellular calcium release through the IP_3_ receptor also contribute to ER stress‐induced apoptosis (Urra et al., 2013). In summary, the UPR consists of a combination of signaling pathways that enforce adaptive reactions to recover ER proteostasis and adjust the folding capacity of the cell, or trigger the activation of apoptosis of an irreversibly damaged cell.

### 1.3 Functional studies linking ER proteostasis with aging in simple model organisms

The UPR is therefore a crucial component of the cell's proteostasis maintenance mechanisms. As cells age, however, their ability to maintain proteostasis is lost, and evidence from simple model organisms suggests that impaired UPR function may contribute to this decline in homeostasis. In *C*.* elegans*, the ability to induce the activation of UPR target genes upon experimental ER stress is abrogated relatively early in the aging process (Ben‐Zvi et al., 2009; Labbadia & Morimoto, 2015; Taylor & Dillin, 2013). This phenomenon reduces the resistance of older animals to stress and seems to result from a loss of the ability to activate the IRE1α/XBP1 branch of the UPR (termed IRE‐1/XBP‐1 in worms) (Taylor & Dillin, 2013). Indeed, mutations in either IRE1α or XBP1 cause shortened lifespan in the worm.

A functional UPR is also necessary for lifespan extension mediated by a variety of pathways in model organisms, suggesting again that it plays a crucial role in longevity assurance. Increased lifespan through reduced insulin/IGF1‐like signaling (IIS) in *C*.* elegans* requires the presence of both IRE1α and XBP1, although the UPR does not appear to be activated in *daf*‐*2* mutant animals in which IIS is reduced (Henis‐Korenblit et al., 2010). In addition, XBP1 is required in yeast for enhanced longevity and gene regulation in conditions of dietary restriction (DR), and IRE1α is required for extension of lifespan by DR in *C*.* elegans* (Chen et al., 2009; Choi et al., 2013). Recent evidence suggests that, in the worm, DR induces a higher basal level of UPR activation and elevated expression of ER protein processing genes, dependent on the DR‐associated transcription factor PHA‐4, and delays age‐associated loss of UPR activation (Matai et al., 2019). In *Drosophila melanogaster*, intestinal IRE1α is required for longer lifespan in dietary restricted animals, by impacting lipid metabolism to promote metabolic adaptation through increased triglyceride synthesis in the intestinal epithelium (Luis et al., 2016). These connections between diet, metabolism, and the UPR seem to be widespread; indeed, a study in *C*.* elegans* exploring the lifespan‐lengthening effects of vitamin D have suggested that, again, the longevity and improved proteostasis induced by this nutrient require the UPR genes IRE1α and XBP1, in a pathway involving the oxidative stress‐related transcription factor SKN‐1 (Mark et al., 2016).

Activation of the UPR can itself be sufficient to increase lifespan. Constitutive activation of the UPR in yeast, through deletion of UPR target genes or other genes that increase UPR activation, extends replicative lifespan (Cui et al., 2015; Labunskyy et al., 2014). In *C*.* elegans*, transient treatment with pharmacological agents that cause ER stress and induce UPR activation, such as tunicamycin, also increases lifespan, in an IRE1α‐dependent manner (Matai et al., 2019). In addition, activating the UPR genetically, through expression of the spliced and active form of XBP1, XBP1s, is also sufficient to extend longevity in the worm (Taylor & Dillin, 2013). Moreover, *Xbp1s* expression also improves proteostasis and stress resistance and protects against toxicity in models of proteotoxic disease (Imanikia et al., 2019). Key to this enhanced longevity and improved proteostasis is the increased acidity and activity of lysosomes in the *C*.* elegans* intestine following intestinal UPR activation, mediated by *Xbp1s*‐induced transcriptional upregulation of lysosomal genes, which increases the clearance of toxic, misfolded protein species (Imanikia, et al., 2019). Intestinal UPR activation also alters the animal's fat storage and fatty acid composition through changes to the activity of fatty acid desaturases and lysosomal lipases, as well as increases in lipophagy, which contributes to enhanced longevity (Daniele et al., 2020; Imanikia et al., 2019). UPR signaling therefore intersects with metabolism and proteostasis to sustain healthspan.

**FIGURE 2:**
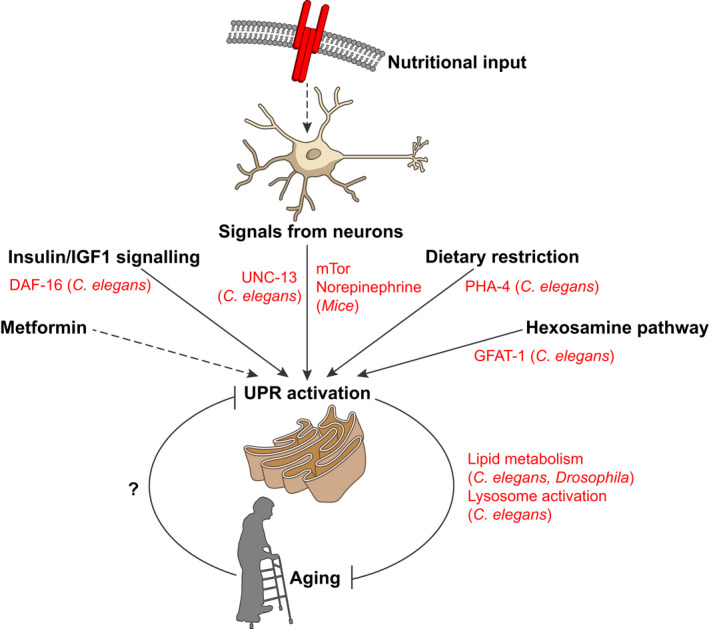
Crosstalk between aging pathways and the UPR. The interrelation between signaling pathways that regulate aging and the UPR is indicated, with key components highlighted in red, in addition to intervention strategies that modify the aging process

The importance of maintaining ER homeostasis during aging means that ER‐related pathways beyond the UPR are also important for longevity assurance. In *C*.* elegans*, increased activity of the hexosamine pathway, which synthesizes *N*‐glycan precursors, induces both ERAD and autophagy, improving proteostasis and stress resistance, and extending lifespan (Denzel et al., 2014). While the UPR is not activated in *gfat*‐*1* animals which have increased hexosamine pathway flux, lifespan extension and proteostasis improvement in these animals depends on the presence of IRE1α and XBP1 and may also involve the ISR (Denzel et al., 2014; Horn et al., 2020). In addition, recent evidence suggests that the cell‐surface hyaluronidase TMEM2, which modulates the composition of the extracellular matrix, also regulates lifespan and aging in both *C*.* elegans* and human cells, through effects on ERK/p38 MAPK pathways that promote enhanced ER stress resistance (Schinzel et al., 2019). The maintenance of ER proteostasis may therefore be a nexus upon which numerous pathways that influence lifespan converge (Figure 2).

### 1.4 The UPR in mammalian brain aging and neurological function

Recent functional studies in mouse models have demonstrated a fundamental role of the ER proteostasis network in determining when and how the brain ages. Retinal damage often occurs in diabetic neuropathy, a pathological condition resulting in vision loss in aged adults. Analysis of the progression of retinal degeneration in a mouse model of type I diabetes indicates that the genetic ablation of XBP1 expression in the retina results in an enhanced disruption of photoreceptor function during aging (McLaughlin et al., 2019). However, XBP1 deficiency alone did not provoke spontaneous alterations to retinal function at basal levels. Aging has been associated with a modification of the sleep and wake quality and quantity. The possible impact of the ER protein folding machinery was study over sleep–awake behavior (Naidoo et al., 2018). Authors took advantage of a BiP heterozygous animal as a strategy to reduce the levels of this abundant and essential ER chaperone in the brain. Data indicated a significant increase in the wake period and also the activity of non‐rapid ocular movements in BiP mutant mice during aging (Naidoo et al., 2018). Consistent with this, a recent report indicated that the eIF2α/ATF4 pathway is central to control of the circadian clock and sleep behaviours (Pathak et al., 2019).

A few interesting studies have suggested that, similarly to the observations reported using *C*.* elegans*, the mammalian UPR is engaged by known intervention strategies that improve healthspan. For example, the positive effects of metformin, an AMPK activator, on age‐dependent hearing loss correlate with a modulation of ER stress levels (Cai et al., 2020). Metformin was also shown to improve cognitive function in a model of accelerated senescence and Alzheimer‐like pathology (SAMP8 mice), involving a reduction in ER stress levels, highlighting an attenuation of PERK signaling (Liu et al., 2020). However, the data provided in those studies are correlative and the functional role of the UPR/ER stress in the protective effects of metformine was not determined.

Although an explosion of studies in the last year using *C*.* elegans* point to a central role of the neuronal UPR in the control of organismal aging, studies in mammals remained highly correlative until this year. It has been reported that the genetic or pharmacological reduction of eIF2α phosphorylation or ATF4/PERK expression improves synaptic plasticity and the performance of animals in memory‐related tasks (reviewed in (Martinez et al., 2018)). Three studies available in the *BioRxiv repository* might provide the first direct evidence for an involvement of the UPR in normal brain aging (Cabral‐Miranda et al., 2020; Krukowski et al., 2020; Longo et al., 2020). Genetic depletion of PERK in dopaminergic neurons was shown to reduce dopamine release in the striatum on an age‐dependent manner, associated with progressive motor dysfunction (Longo et al., 2020). In contrast, treatment of aged animals with ISRIB, a small molecule that blocks the consequences of eIF2α phosphorylation, reverses the natural decay of cognitive function in mice, improving behavioral, and electrophysiological parameters (Krukowski et al., 2020). Finally, genetic deletion of IRE1α in the mouse brain accelerates age‐dependent decline in motor and cognitive functions, negatively impacting hippocampal function (Cabral‐Miranda et al., 2020). IRE1α deficiency in the brain did not affect the performance of young animals, suggesting the occurrence of age‐dependent phenotypes. Remarkably, the artificial expression of the active form of XBP1s in the brain using transgenic mice prevented age‐dependent decline of brain function. And, importantly, gene therapy to specifically deliver XBP1s into the hippocampus was able to revert the spontaneous decline of learning and memory capacity, thus restoring cognitive function in aged animals (Cabral‐Miranda et al., 2020).

Importantly, the genetic manipulation of the IRE1α/XBP1s pathway in the brain altered the content of senescent cells, one of the main players driving aging. Thus, these studies suggest that ER proteostasis is disrupted during mammalian brain aging and that strategies to artificially boost the capacity of the UPR might prevent or delay the normal decline of brain function as we age.

### 1.5 ER proteostasis in human aging

The field has advanced in the last year to provide interesting correlations between human tissue aging and the presence of ER stress markers. For example, a recent study indicated a correlation between aging and the attenuation of UPR signatures in human muscle samples from healthy subjects (Hart et al., 2019). The transcriptomic profiling of muscle biopsies from a small group of people (*N* = 12) of an average 27 years of age compared to older subjects with an average age of 75 years indicated modest changes in the activity of the UPR at resting conditions (Hart et al., 2019). However, analysis of muscle samples 18 h after exercise indicated a marked upregulation of diverse components of the ER proteostasis network (i.e., ATF3, PERK, GADD34, BiP, XBP1) in the muscle of young people, whereas this induction was attenuated in older subjects (Hart et al., 2019). In addition, a correlation between UPR activation, the modulation of autophagy markers, and signs of satellite cell differentiation was provided in the study.

ER stress is a major component contributing to ALS progression (Rozas et al., 2017). A recent report monitored the levels of ATF6 activation in peripheral mononuclear cells derived from patients suffering sporadic ALS (Prell et al., 2019). Interestingly, in addition to providing a correlation between ALS diagnosis and the activation of ATF6 processing in blood cells, a progressive rate of activation of ATF6 was observed only in ALS patients of younger age, versus a stable activation observed in older patients, suggesting a reduction in the buffering capacity of the UPR (Prell et al., 2019). Finally, another study analyzed the levels of ER stress markers in postmortem human eye lens derived from people ranging from 50 to 90 years of age (Tang & Yang, 2015). Remarkably, a progressive and almost linear increase in the levels of BiP, total ATF6, and IRE1α levels were observed in human lenses as aging advances (Tang & Yang, 2015). Thus, emerging evidence suggests an association between aging and alterations to ER proteostasis components in human‐derived tissue.

### 1.6 Cell‐nonautonomous control of the UPR

**FIGURE 3:**
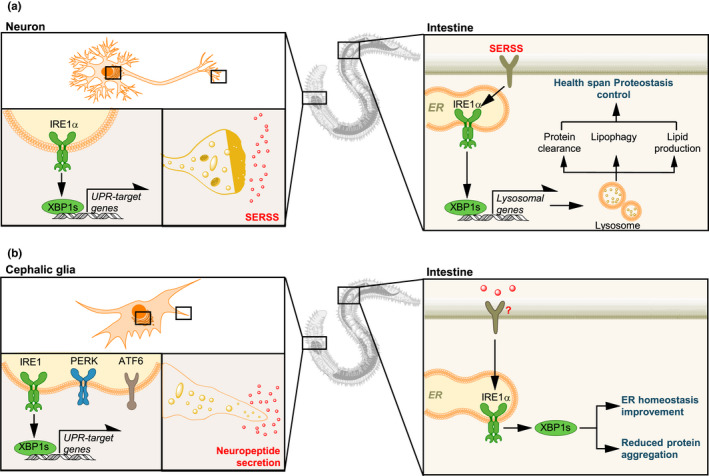
Cell‐nonautonomous control of the UPR in aging. (a) In *Caenorhabditis* *elegans* expression of XBP1s in neurons signals to distal tissues (i.e., the intestine) to activate IRE1/XBP1 in a cell‐nonautonomous manner, driving proteostatic changes that are central to lifespan and healthspan (ER proteostasis effectors, lipid metabolism, and lysosomal function). An unknown secreted ER stress signal (SERSS) may mediate the communication between neurons and the gut to engage the UPR. (b) In addition, expression of XBP1s in cephalic glia of *C*.* elegans* also increases life span, independently of neurons, engaging the same UPR signaling branch in the gut. This process depends on neuropeptide release

One major surprise that has emerged from analysis of the role of the UPR in aging has been the discovery that UPR activation can be transmitted between tissues, leading to cell‐nonautonomous induction of this stress response in cells that have not experienced stress (Figure 3) (Taylor et al., 2014). In *C*.* elegans*, this communication was first identified between the neurons of the worm and its intestine; mutations in a neuronal receptor can induce UPR activation in distal tissue, and expression of active, spliced *Xbp1s* in the nervous system leads to intestinal UPR activation that in turn improves ER stress resistance, enhances proteostasis, and increases lifespan (Imanikia, et al., 2019; Sun et al., 2011, 2012; Taylor & Dillin, 2013). This communication between neurons and the intestine depends upon the release of neuronal factors that are governed by the secretory regulator UNC‐13 and results in the improved intestinal lysosome activity and enhanced lipid metabolism as already discussed (Figure 3a). The identity of the signaling mediator(s) in this system, however, remains unclear.

A similar pathway may exist in mice. Expression of XBP1s in the proopiomelanocortin (POMC) neurons of the murine hypothalamus leads to upregulation of UPR targets in the liver, in a manner analogous to the cell non‐autonomous UPR activation seen in worms (Williams et al., 2014). This leads to higher energy expenditure and resistance to diet‐induced obesity, independent of food intake, as well as increasing insulin sensitivity, resulting in better glucose regulation. Indeed, the neuronal perception of food can itself induce UPR activation in the liver, which is accompanied by phosphatidylcholine synthesis and ER remodeling (Brandt et al., 2018). Food perception or the optogenetic activation of POMC neurons is sufficient to promote hepatic norepinephrine signaling, followed by mTOR and XBP1 activation within hepatocytes, priming the liver for food consumption. Again, the connection between UPR activation and metabolic adaptation seems to be of prime importance. It is not yet clear, however, whether this pathway can also influence longevity.

Cell‐nonautonomous UPR activation within the intestine has also been observed in *Drosophila melanogaster*, regulating tissue homeostasis within the intestinal epithelium. Aging leads to over‐proliferation of intestinal stem cells (ISCs) within the barrier epithelium of the fly gut. In this context, strategies to improve ER homeostasis through expression of *Xbp1s* or activation of ERAD, can reduce age‐associated overgrowth in the gut, extending lifespan (Biteau et al., 2008, 2010; Wang et al., 2014). PERK activation, however, has the opposite effect, and knockdown of PERK both improves intestinal homeostasis and extends lifespan in flies (Wang et al., 2015). In fact, PERK activation and eIF2α phosphorylation, followed by ISC proliferation, can be mediated by ER stress‐induced in entirely different cells, the enteroblasts or enterocytes. These findings suggest that cell‐nonautonomous activation of PERK occurs between these cell types and the ISCs (Wang et al., 2015). This communication of UPR activation depends on the induction of inflammatory cytokines through JNK signaling, suggesting a mechanism by which cell‐nonautonomous UPR activation and subsequent effects on tissue homeostasis and lifespan may operate in this species.

Other cell types have also been implicated as instigators and receivers of cell non‐autonomous UPR‐activating signals. Recent evidence suggests that glia, for example, may act as mediators of cell non‐autonomous UPR activation (Figure 3b). Work in *C*.* elegans* has demonstrated that, as in neurons, constitutive expression of *Xbp1s* in glia leads to distal activation of the UPR in intestinal cells (Frakes et al., 2020). This UPR activation in the cephalic sheath glia, and subsequent distal UPR activation, improves proteostasis and extends longevity. However, unlike the corresponding neuronal pathway, in which the secretory regulator UNC‐13 but not the neuropeptide‐specific regulator UNC‐31 is required for signaling, downstream UPR activation seems to depend upon UNC‐31‐dependent neuropeptide release, suggesting that the glial route to intestinal UPR activation operates through a different mechanism. Given that activation of the PERK‐eIF2α UPR branch in murine glia can also affect the glial secretome, with downstream effects on other cells—including modulation of neuronal synaptic connections and neurodegeneration—this raises the intriguing possibility that UPR activation and consequent secretory changes in glia may also mediate UPR‐activating effects that might influence longevity in mammalian systems (Smith et al., 2020).

In addition, studies in human cell culture systems have suggested that conditioned medium from ER‐stressed tumor cells can activate the UPR in downstream immune and liver cells, leading to pro‐inflammatory responses in macrophages and dendritic cells—although the veracity of this signaling mechanism has been disputed (Mahadevan et al., 2011; van Ziel et al., 2020). In tumors themselves, activation of the UPR through factors that include hypoxia and nutrient starvation can lead to “anticipatory” UPR activation in neighboring tumor cells, a process that involves the release of steroid and peptide hormones (reviewed in (Shapiro et al., 2016)). In receiving tumor cells, this engages downstream IP_3_R calcium channels and results in phosphorylated phospholipase Cg (PLCg) activation, UPR activation, and enhanced cell survival. These findings suggest that cell‐nonautonomous UPR activation may represent a conserved and even widespread phenomenon, that may impact upon cellular health and survival in mammals similarly to invertebrate model organisms.

## 2 PERSPECTIVE

Aging is the main risk factor to develop most chronic diseases affecting the human population. Among the biological pillars of aging, a decline in the buffering capacity of the proteostasis network is emerging as a fundamental process altered during aging, where the ER is a relevant node of this network that functionally contributes to the aging process across species. Because protein misfolding and aggregation is a salient feature of a variety of age‐related diseases (i.e., neurodegeneration, diabetes, cancer, fibrosis, systemic amyloidosis among others), it might be feasible to speculate that strategies to improve ER proteostasis in the aging population might reduce the risk to develop these diseases.

Many different reports have shown the presence of markers of chronic ER stress in diverse tissues in mammals (i.e., upregulation of BiP and Chop, and other UPR target genes), including brain, bond, pancreas, gut, muscle, and eye (reviewed in (Martinez et al., 2017)). These results are in conflict with the idea that the activity of the UPR, and more specifically the IRE1α/XBP1 branch, is attenuated during aging. However, experimental induction of ER stress with pharmacological agents has been shown to result in attenuated UPR signaling in the aging brain compared to young animals (Cabral‐Miranda et al., 2020). It might be possible that the inability to properly engage the UPR in the long‐term produces damage to ER physiology that is compensated for by the upregulation of proteostasis components (ER stress markers) through alternative regulatory pathways. Alternatively, sustained and chronic ER stress may downregulate the response by feedback mechanisms that attenuate the UPR, disabling further responses to exogenous pharmacological stress.

As discussed, accumulating studies using *C*.* elegans* suggest that the activity of the UPR in neurons has a key role in adjusting global proteostasis at the whole organism level, where the connection between the brain and the gut operates as a central regulator of the aging process. Many questions remain open in the field (Box 1). Does brain UPR regulate mammalian aging through a connection with the intestine? This may be feasible because the activation of the UPR in a cell‐nonautonomous manner has been reported in mammals, where the activity of XBP1 in the hypothalamus mediates the engagement of mirror responses in peripheral tissues. Since the hypothalamus is a relevant component in the regulation of the brain and gut axis (Frankiensztajn et al., 2020), and both organs are functionally connected, it remains an open question whether the beneficial effects of activating XBP1 in the brain are mediated in part through an improvement of intestinal physiology. Of note, the expression of IRE1α/IRE1β and XBP1s have relevant functions in sustaining gut physiology, where the genetic disruption of the pathway results in higher susceptibility to develop colitis and generates increased inflammation (Kaser et al., 2013), consistent with the fact that a polymorphism in the XBP1 gene increases the risk to develop inflammatory bowel diseases (Kaser et al., 2008). Thus, if neuronal XBP1s signals to the intestine to engage the UPR, it is expected to result in protection against age‐dependent gut dysfunction. In this scenario, since gut microbiota alterations contribute to neurological conditions such as autism and Parkinson's, fine tuning the UPR in the brain promises interesting therapeutic avenues to improve brain function during aging through the gut or gut microbiota.

Box 1 **Outstanding questions**

How does aging lead to a loss of UPR activation?

Which molecules mediate cell‐nonautonomous UPR activation in different species?

Does a brain‐to‐intestine UPR activation axis exist in mammals?

How does UPR activation influence mammalian aging?

Is the UPR a potential target to treate neurodegenerative disease?

Another important question to be solved is what are the signals that engage the UPR in the periphery when the pathway is activated in neurons. One possibility is that signaling events (neuronal connectivity or the release of soluble factors) activate UPR sensors in an ER stress‐independent manner. In agreement with this idea, many reports have shown that the UPR plays central roles in a variety of physiological processes where stress sensors are engaged in an ER stress‐independent manner, including cytokine production in macrophages, B cell differentiation, angiogenesis, cell migration, mitochondrial metabolism, and BDNF production, among other functions. However, it might be possible that neuronal UPR activation simply triggers the activation of cellular processes that impose a demand on the secretory capacity of intestinal cells, resulting in physiological levels of ER stress. All these questions remain to be solved, representing fundamental goals in the drive to understand the mechanisms that explain the beneficial effects of the UPR during aging. Although indirect evidence has linked the UPR with canonical pathways driving the aging process (i.e., senescence, IGF signaling, inflammation), more studies are needed to define how ER stress signaling, or the activity of the secretory pathway, is directly linked to the hallmarks of aging. Interestingly a recent report directly implicated the activity of IRE1α/RIDD in the downstream modulation of the DNA damage response affecting DNA repair and cell cycle control (Dufey et al., 2020), a pathway that has been extensively connected with the process of senescence. In addition, genetic targeting of XBP1 in the mouse brain was recently shown to trigger protective compesatory events resulting in the upregulation of IGF2, improving proteostasis in models of Huntington's disease (García‐Huerta et al., 2020). Because many small molecules (Hetz et al., 2019) and gene therapy approaches (Valenzuela et al., 2018) are under development to tune the ER proteostasis network in a disease context, interesting opportunities may become available in the near future to target the UPR in the context of aging, which promise an interesting holistic approach to prevent or attenuate the emergence of a variety of age‐related diseases affecting the human population.

## CONFLICT OF INTEREST

The authors declare no competing interests.
